# Improving the Integration between Palliative Radiotherapy and Supportive Care: A Narrative Review

**DOI:** 10.3390/curroncol29100627

**Published:** 2022-10-19

**Authors:** Erica Scirocco, Francesco Cellini, Costanza Maria Donati, Jenny Capuccini, Romina Rossi, Milly Buwenge, Luigi Montanari, Marco Maltoni, Alessio Giuseppe Morganti

**Affiliations:** 1 Radiation Oncology, IRCCS Azienda Ospedaliero-Universitaria di Bologna, 40138 Bologna, Italy; 2 Department of Experimental, Diagnostic and Specialty Medicine, Alma Mater Studiorum University of Bologna, 40138 Bologna, Italy; 3 UOC di Radioterapia Oncologica, Dipartimento di Diagnostica per Immagini, Radioterapia Oncologica ed Ematologia, Fondazione Policlinico Universitario “A. Gemelli” IRCCS, 00185 Roma, Italy; 4 Istituto di Radiologia, Università Cattolica del Sacro Cuore, 00185 Roma, Italy; 5 Palliative Care Unit, AUSL Romagna (Local Health Authority), 48022 Lugo, Italy; 6 IRCCS Istituto Romagnolo per lo Studio dei Tumori (IRST) “Dino Amadori”, 47014 Meldola, Italy; 7 Medical Oncology Unit, IRCCS Azienda Ospedaliero-Universitaria di Bologna, 40138 Bologna, Italy

**Keywords:** literature review, radiotherapy, palliative, supportive care, training, organizational models

## Abstract

Palliative radiotherapy (PRT) is known to be effective in relieving cancer related symptoms. However, many studies and clinical practice show several barriers hindering its use and worsening the quality of patient support during PRT. Various solutions were proposed to overcome these barriers: training on PRT for supportive and palliative care specialists and training on palliative care for radiation oncologists, and introduction of pathways and organizational models specifically dedicated to PRT. Evidence on innovative organizational models and mutual training experiences is few and sparse. Therefore, the aim of this literature review is to present a quick summary of the information available on improving the PRT quality through training, new pathways, and innovative organizational models. The majority of studies on the integration of PRT with other palliative and supportive therapies present low levels of evidence being mostly retrospective analyses. However, it should be emphasized that all reports uniformly showed advantages coming from the integration of PRT with supportive therapies. To actively participate in the integration of PRT and palliative care, providing comprehensive support to the needs of patients with advanced cancer, radiation oncologists should not only plan PRT but also: (i) assess and manage symptoms and stress, (ii) rapidly refer patients to specialists in management of more complex symptoms, and (iii) participate in multidisciplinary palliative care teams. To this end, improved education in palliative care both in residency schools and during professional life through continuous medical education is clearly needed. In particular, effective training is needed for radiotherapy residents to enable them to provide patients with comprehensive palliative care. Therefore, formal teaching of adequate duration, interactive teaching methods, attendance in palliative care services, and education in advanced palliative care should be planned in post-graduated schools of radiotherapy.

## 1. Introduction

Palliative radiotherapy (PRT) is effective in relieving cancer related symptoms, with few side effects due to the low radiation doses and low treatment-related costs.

However, many studies and clinical practices highlight a number of barriers hindering the use of PRT. Among these, the following were identified: difficulty in traveling to reach a radiotherapy center for patients with advanced cancer [1,2,3], concern about the need for prolonged therapies [4,5], delay in consultations and treatment, complicated referral process, difficulty in contacting radiation oncologists (ROs) [6], difficulty in predicting prognosis [7] with consequent risk of PRT delivery near the end of life [8], poor knowledge of PRT by specialists in palliative care [9,10,11,12], poor training of ROs in palliative care [13,14,15,16] with consequent feeling of inadequacy in managing patients in the complex setting of advanced cancer, and complex logistical scenarios such as those imposed by the COVID-19 pandemic [17]. 

Several solutions have been proposed to overcome these barriers including: the use of tools to improve prognosis prediction [18], training on PRT for experts in palliative care [9,10,11] and on palliative care for ROs [13,14,15,16], and introduction of pathways and organizational models [19,20,21,22,23], possibly integrated with supportive care programs, specifically dedicated to PRT [24,25,26,27,28,29,30,31,32,33,34,35,36]. In particular, PRT rapid response programs in which patient assessment and radiotherapy planning and delivery are performed in the shortest time (possibly on the same day) were proposed [37,38,39]. However, if an in-depth literature is available on the organization of PRT rapid response programs, the evidence on organizational models and mutual training experiences is less and sparse.

Therefore, the aim of this literature review is to present a quick summary of the information available on improving the PRT quality through training and new pathways and organizational models.

## 2. Methods

This narrative review of the literature was performed by a multidisciplinary team of ROs and palliative care specialists using the PubMed, Scopus, and Cochrane library bibliographic databases. Papers on innovative organizational models and educational projects on PRT were included in the research. The search was initially performed in September 2021 and subsequently updated in April 2022, using various combinations of the following terms: palliative, supportive care, radiotherapy, hospice, integration, training, and organizational models. Only papers in English were included in this review, with no time limits. The selection of the papers to be included in the review was carried out independently by two authors (ES and JC) and any discrepancies were resolved by consulting the senior author (AGM). The extraction of information from the selected papers was performed independently by two other authors (CMD and MB) and any discrepancies were resolved through discussion. The list of references of the selected papers was consulted in order to identify other publications relevant to the purposes of the review. Overall 51 abstracts and 38 papers in full were consulted. Of the latter, 16 were excluded for multiple reasons: some studies were descriptions and did not reported specific data, others did not focused on our aim, and two were case reports. In conclusion, 22 papers were included in this narrative review.

## 3. Results

The initial search on bibliographic databases led to the identification of 188 papers, potentially useful for the purposes of this analysis. The examination of the titles and abstracts led to the selection of 54 papers that were examined in full-text, as 113 papers were found to be irrelevant to the subject of the review, 5 papers were published in a language other than English, and 16 papers were editorial or review. Of the papers evaluated in full-text, 22 were selected and included in this review while 32 papers were considered not relevant to the topic of this analysis.

### 3.1. Improving Palliative Radiotherapy

Some studies tested the potential utility of establishing radiotherapy units or services specifically dedicated to PRT (Table 1). 

Chang et al. described a multidisciplinary service model within a radiotherapy department (Palliative Radiation Oncology Consult—PROC) and tested the effects of this dedicated unit with propensity score adjusted analysis. The study showed that after the introduction of the PROC: (i) single fraction treatments were more frequently used, (ii) the duration of hospitalizations was reduced, (iii) the use of specialty level palliative care increased significantly [19]. Furthermore, Tseng et al. reported an analysis of the satisfaction of the ROs in charge of palliative treatments offered by a service dedicated only to PRT (Supportive and Palliative Radiation Oncology—SPRO). The analysis showed a significantly higher degree of satisfaction compared to that expressed by ROs operating in radiotherapy departments without a service dedicated to PRT. Moreover, Job et al. analyzed the impact of a referral pathway specifically dedicated to patients undergoing PRT (Palliative Advanced Practice Radiation Therapist). The results of their analysis showed that, compared to the standard pathway, patients in the dedicated pathway had a shorter waiting time for PRT initiation and completed the PRT with fewer hospital visits [23]. Finally, LeGuerrier et al. reported the results of an analysis that indirectly suggested the successful integration between supportive care and PRT. In fact, an integrated service was initially planned (symptoms management at the same time as evaluation for PRT) with a staff of two people working half a day a week. However, after 11 years the service was based on a staff of six members working five full days a week [22].

### 3.2. Palliative Radiotherapy Integration with Other Palliative or Supportive Treatments

In this paragraph we report on the experiences of integration between PRT and supportive or palliative therapy services. The results are detailed in the second subparagraph and summarized in Table 2, while the first subparagraph shows, as an introduction to the second, the results of some studies on real world data about what happens when the integration between PRT and supportive therapies it is not realized.

#### 3.2.1. Real World Scenarios

Some studies investigated the use of palliative care in patients undergoing PRT and of PRT in patients managed in palliative care setting.

A retrospective analysis conducted in the US showed that only a minority of patients undergoing PRT also benefit from palliative care services. The authors commented that this scenario should be modified with appropriate educational interventions for ROs [27]. Conversely, Al-Shahri et al. analyzed the referral to PRT in patients admitted to a palliative care unit. The analysis, conducted in Saudi Arabia, showed that out of 635 patients hospitalized over four years only 25 (3.9%) were referred to PRT. Two ROs rated their referrals as adequate and the authors concluded that, in the setting of inpatients with advanced cancer, PRT is scarcely used [30]. Eastman et al. reached similar conclusions in their study conducted in Australia. In fact, from the patients admitted to a regional palliative care unit, only 3% were treated with PRT. Furthermore, the author reported 32–42% reduction in opioid drug use in irradiated patients [31]. 

#### 3.2.2. Integrating Palliative Radiotherapy in Multidisciplinary Patients Management

Unlike those just mentioned, other studies reported on organizational-care models based on the integration of PRT within a multidisciplinary management of patients or on PRT models aimed at reducing hospitalization and favoring home care or outpatient status.

In terms of integrating PRT into a multidisciplinary management of cancer patients, Manfrida et al. reported the results of the IMMA study (IMprovement in MAnagement of pain) on a multidisciplinary management program (ROs and anesthetists) of pain. The team met weekly to evaluate drug therapy and the possible indication for PRT in cancer patients with pain. The authors observed that, four weeks after evaluation and subsequent interventions, the percentage of patients with inadequate pain management decreased from 27.7% to 1.5% [36], and that, at 34 weeks, patients treated with RT had complete and partial response of 28.8% and 46.7%, respectively; pain progression and indeterminate response were 0.95% and 23.8%. Moreover, Jung et al. described the results of an integrated management model of patients with brain metastases by a multidisciplinary team including palliative care and radiotherapy services as well as nurses and other healthcare professionals [33]. From a total of 100 evaluated patients, 75 underwent PRT, and only 9% of them died within 30 days of therapy. An advance care planning discussions was taken in almost 57 patients at first consult visit. Overall survival was consistent with literature reports. Based on this result, the authors observed that a multidisciplinary management of this complex care setting improves the proper use of PRT. Furthermore, Pituskin et al. systematically referred all patients undergoing PRT planning for bone metastases to a multidisciplinary evaluation involving pharmacists, dieticians, occupational therapists, and social workers. The study showed that the patients evaluated by the team received a greater number of prescriptions and recommendations useful for their clinical management. They also reported higher rates of relief not only from pain but also from fatigue, anxiety, and depression [34]. Moreover, Nider et al. published the results of two studies comparing patients undergoing PRT alone with subjects managed by a multidisciplinary palliative cancer care team. The first analysis showed that there were no significant differences in survival between the two different types of care. Therefore, the authors concluded that the impact of multidisciplinary palliative care should be investigated prospectively on other relevant endpoints such as symptom control, side effects, and quality of life [24]. Similarly, in the second study, the authors compared patients undergoing PRT as part of normal clinical practice with subjects managed as part of a multidisciplinary palliative care team. Despite the fact that the second group included significantly more patients with reduced performance status and with higher Edmonton symptom assessment system symptom scores (particularly in terms of pain, fatigue, anxiety, and depression), the actuarial overall survival was comparable among the two patients populations and also the rate of PRT during the last month of life was comparable [25]. Finally, Spedicato et al. reported on an Italian home care experience, which began in the 1990s, for terminally ill cancer patients receiving supportive care by a team of ROs, nurses and psychologists. When needed, the team received support from anesthetists and pain therapists. Moreover, patients were treated with PRT, by the same ROs who took care of them at home, during short stays in the radiotherapy department. A result from this experience was the clear appreciation, by patients and family members, of the continuous care by the same team both at home and in hospital [35]. (Table 2).

#### 3.2.3. Improving Home Care and Outpatient Status by Palliative Radiotherapy

In terms of shorter duration of hospitalization, Ishii et al. used PRT, possibly integrated with other supportive treatments, in elderly patients (>75 years) with advanced esophageal cancer, in order to allow for outpatient clinical management. Thirty-six patients were treated with PRT and the median overall and outpatient survival was 14 and 9 months, respectively. The authors concluded that, in the palliative treatment of esophageal cancers, PRT may allow for prolonged outpatient management [32]. Subjects with esophageal cancer extending all or most of the circumference of the organ required additional medical procedures. Moreover Cellini et al. proposed a clinical care model (NORMALITY) developed to reduce the logistical limitations imposed by the COVID-19 pandemic but subsequently aimed at shortening the length of hospital stays for patients who need PRT but who have to face complex logistical contexts (need for home care or hospice, living far from the nearest radiotherapy center). The aim of the model was to perform radiotherapy consultation, simulations and first (and possibly single) PRT fraction on the same day. The pathway is based on two levels of tele-consultation (triage and remote visits) before the patient visits in the triage phase. Some basic information collected by a doctor or a nurse is sent to the radiation therapy service. Then, if needed for PRT planning, the triage can be followed by one or more remote patient assessments by interactive video calls, and the possibility of images sharing [17] (Table 3).

### 3.3. Educational Needs

#### 3.3.1. Needs of Hospice and Palliative Medicine Physicians

Some studies reported on training needs on radiotherapy for hospice and palliative medicine physicians. In particular, two interesting analyzes on this topic have been published by researchers from the University of California [9,10,11].

In the first report, Martin et al. published in 2019 an analysis on implementing a training course about PRT for fellows in hospice and palliative medicine. The course included a formal teaching phase (three lessons of one hour each) and a visit to a radiotherapy service. After the course, the fellows reported their perception of better knowledge of radiotherapy and more favorable attitude regarding: (i) referring patients to PRT, (ii) collaborating with ROs, (iii) considering ROs as necessary professionals in a multidisciplinary palliative team [9]. The following year, Martin and Jones published the results of a survey involving hospice and palliative medicine fellows on their knowledge on PRT. Almost all fellows stated that PRT principles should be included in palliative care training and 75% of them stated that a better understanding of radiotherapy would facilitate their referral of patients to PRT and their collaboration with ROs. Furthermore, fellows trained on PRT presented a more favorable attitude towards PRT, especially if their training on the subject lasted at least five hours [11]. The same year, Martin and Jones published another survey on PRT education among fellowship program directors. Approximately all directors stated the importance of a PRT curriculum in hospice and palliative medicine fellowship programs. However, PRT curriculum is absent in 30% of their programs, and only 14% have more than two hours of PRT education. Finally, more than 75% of directors indicated that they would consider PRT curriculum in their programs [10] (Table 4).

#### 3.3.2. Needs of Radiation Oncologists and Residents in Radiotherapy

Conversely, other studies investigated the training needs of ROs and residents in radiotherapy. In particular, the results of three surveys conducted in the USA by the same group of researchers are particularly interesting [14,15,16].

Krishnan et al. reported the results of a survey involving residents in radiotherapy on their perception of the importance of acquiring clinical skills in palliative care and their judgment on the adequacy of their training. Majority of the participants stated that this topic is very important, but that their training was inadequate. The authors concluded that palliative care education in radiotherapy residency schools should be improved [14]. Furthermore, Wei et al. published the results of a second survey involving the directors of radiotherapy specialization schools in the USA. The analysis showed that only 67% of training programs included topics on palliative and supportive therapies and that only in a minority of schools did the curriculum include topics such as fatigue, spiritual issues, and advanced palliative care programs. Furthermore, teaching on PRT was focused on bone and brain metastases, with only hints on skin and visceral metastases. The authors concluded that also this analysis showed clear room for improvement [15]. Finally, the same authors further reported the results of a third survey involving US ROs and concerning their perception of their ability in supportive and palliative care. The study showed that ROs felt they were sufficiently skilled in pain management but with little experience in the treatment of fatigue, anorexia, anxiety, and depression. The main concern of ROs were: (i) fear of bothering referring medical oncologists; (ii) lack of time during the visit; (iii) need to initiate discussions with patients and their families about care planning. In this case, the authors concluded that the continuing medical education of ROs on palliative care should be optimized [16]. 

Instead, Lo Presti et al. published the results of a systematic literature review on the perception of ROs and trainees in radiotherapy on the role, training level and needs in the field of palliative care. The analysis found that virtually all ROs consider palliative care to be important along with PRT. Furthermore, more than half of the ROs stated that: (i) the ability of ROs in palliative care is important, (ii) an improvement in the training of residents in this field and a better training of ROs in communicating with patients and in management of pain are needed, (iii) training of ROs in palliative care is inadequate. Moreover, less than half, but more than 20% of ROs stated that: (i) research on PRT and presentation of palliative care topics in radiotherapy meetings are insufficient, (ii) adequate guidelines on palliative care are lacking, (iii) the role of ROs in multidisciplinary teams on palliative care is important but their involvement is poor. The authors concluded that better integration of PRT into palliative multidisciplinary teams requires further efforts to improve training, research, and scientific communication on these issues in the field of radiotherapy [12] (Table 5).

## 4. Summary and Conclusions

In this narrative review we have provided a concise overview of the evidence in the literature on improving the quality of palliative radiotherapy through integration with supportive care. This analysis has obvious limitations: the retrospective design of most of the included reports, the inhomogeneity of the methods and endpoints, and the consequent inability to perform any quantitative analysis (Table S1). Moreover, in most analyzed studies no details on dose and fractionation of the PRT were reported. Only Nieder et al., in their comparisons between PRT alone or integrated in multidisciplinary management, reported that PRT was delivered with hypofractionated regimens (from 8 Gy × 1 fraction to 3 Gy × 10 fractions), [24,25] while Ishii et al., in their series of esophageal tumors, used a conventional fractionation (40–60 Gy in 2 Gy/fraction) [32]. However, it should be emphasized that all reports uniformly showed some advantages from integrating PRT with supportive care.

In fact, the results of the studies on the potential utility of establishing radiotherapy units or services specifically dedicated to PRT, summarized in Table 1, suggest that specifically dedicated services can improve adequacy and timelines of PRT and team members satisfaction [19,20,22,23]. Moreover, some studies on common clinical practice scenarios suggest that mutual patient referral between PRT and palliative care can be very poor without specific interventions [30,31]. However, other reports suggest that integrating PRT into multidisciplinary management of cancer patients may improve the adequacy of pain and other symptoms treatment, the appropriate use of PRT, and patient and family satisfaction [24,25,27,33,34,35,36] (Table 2). Furthermore, especially if planned with innovative organizational models, PRT can promote outpatient or home care management [17,32] (Table 3). Finally, the results of the studies on education and training needs, summarized in Table 4 and Table 5, suggest that improved multidisciplinary collaboration between ROs and palliative care physicians, and consequently the ability to provide high quality palliative care, require mutual training and updating effort.

These evidences suggest that ROs should actively participate in the integration of PRT and palliative care and provide comprehensive support to the needs of patients with advanced cancer. To this end, they should be able not only to correctly plan PRT but also to: (i) assess and manage symptoms and stress, (ii) timely refer patients to specialists in the management of more complex symptoms, and (iii) participate in multidisciplinary palliative care teams [25]. Therefore, there is a clear need to improve the training in palliative care both in residency schools and during professional life through continuous medical education.

In particular, effective training is needed for residents in radiotherapy to enable them to provide patients with comprehensive palliative care in their future professional path. Therefore, they would need a formal training of adequate duration, innovative-interactive teaching methods, attendance in palliative care services, and education on advanced palliative care [23]. 

In conclusion, the majority of the studies on the integration of PRT with other palliative and supportive therapies present low levels of evidence, being retrospective analyses in most cases. However, all reports uniformly showed clear advantages from integrating PRT with supportive care.

## Figures and Tables

**Table 1:** Characteristics and main findings on studies on radiotherapy services dedicated to palliative radiotherapy.

Authors, Year, Center (Ref.)	Setting	Aim	Main Findings
Tseng Y.D., et al., 2014, four US academic centers [20]	Online survey (102 RT care provider)	to evaluate Supportive and Palliative Radiation Oncology’s impact on PCC quality and compare PCC rating among physicians practicing with and without a dedicated PRT service	The majority of RT care providers stated that Supportive and Palliative Radiation Oncology service improves overall quality of care, communication with patients and families, team experience, time spent on technical aspects of PCC, adherence to treatment recommendations and dose/fractionation guidelines, and follow-up
Job M., et al., 2017, Radiation Oncology Mater Center in Brisbane, Australia [23]	Palliative Advanced Practice Radiation Therapist service	to assess if Palliative Advanced Practice Radiation Therapist service improves access to PRT and reduces time from referral to treatment (48 patients were referred to the service)	Patients referred via the Palliative Advanced Practice Radiation Therapist service had a mean and median wait time of 3.5 and 3 days, respectively compared with 8.1 and 5 days for patients referred without it. Patients were also more likely to complete treatment with less visits to the hospital
Chang S., et al., 2018, Mount Sinai Hospital [19]	Palliative Radiotherapy Department	to assess Palliative Radiation Oncology Consult service and compare outcomes between control (154 patients) and post-intervention group (296 patients), before and after the Palliative Radiation Oncology Consult service’s establishment (Observational cohort study)	After establishment of the Palliative Radiation Oncology Consult service: (1) single-fraction (RR: 7.74, 95% CI: 3.84–15.57) and hypofractionated RT (RR: 10.74, 95% CI: 5.82–19.83) was used more frequently; (2) patients required shorter hospital stays (21 vs. 26.5 median days in pre-PROC group), (3) patients were treated with more specialty-level palliative care (OR: 2.65, 95% CI: 1.56–4.49), (4) symptom control was similar (OR: 1.35, 95% CI: 0.80–2.28)
LeGuerrier B., et al., 2019, Cross Cancer Institute in Alberta, Canada [22]	Electronic survey; PRT department	to assess palliative radiation therapists’ experience after 11 years of work in a service dedicated to PRT (7 palliative radiation therapists investigated)	Three palliative radiation therapists were involved one half-day per week for single-fraction PRT in the treatment of symptomatic bone metastases. Afterwards, the model gradually evolved to four palliative radiation therapists, five full clinic days per week

*Legend*: PCC: palliative cancer care; PRT: palliative radiotherapy; RT: radiotherapy.

**Table 2:** Characteristics and main findings on studies about integration of palliative radiotherapy in multidisciplinary patients management.

Authors, Year, Center (Ref.)	Setting	Aim (Study Design)	Main Findings
Spedicato M.R., et al., 1999, Catholic University of the Sacred Heart, Rome, Italy [35]	Home care	to describe a multidisciplinary organizational model of PCC providers dealing with terminally ill patients. Home care and PRT were both managed by the same team of ROs. The team also included psychologists and nurses, with the external support from other health specialists	Holistic care and empathy in the patient’s journey is fundamental. PCC specialists play fundamental roles at every step of the way, especially when working with terminally ill patients
Pituskin E., et al., 2010, Cross Cancer Institute, Canada [34]	Outpatient PRT clinic	to describe the impact of multidisciplinary assessment in patients with bone metastases, treated with PRT, of symptoms, medications, nutritional intake, daily life activities, and psychosocial and spiritual needs. Four weeks after RT, teleconsultation was undertaken to assess improvements (prospective)	Multidisciplinary assessment provide high number of recommendations and decreased symptom distress
Jung H., et al., 2013, Tom Baker Cancer Centre, Canada [33]	Multidisciplinary clinic for RT and supportive care	to assess the impact of a new integrative consultation clinic of PCC, RT, and allied health professionals in 100 patients with brain metastases (Quality assurance study)	75 patients underwent brain PRT, whereas 25 did not (main reasons: patient preference and poor performance status). End-of-life brain RT was in 9% (death within 30 days) and 1% (within 14 days)
Nieder C., et al., 2015, Nordland Hospital, Bodø, Norway [24]	Comparison of 2 groups of patients managed by a multidisciplinary PCC team vs. oncologic staff (29 patients each)	to assess survival after early PRT in patients managed exclusively by regular oncology staff or by a multidisciplinary palliative care team in addition (Retrospective matched pairs analysis)	Median survival was not significantly different at multivariable analysis. Performance status and liver metastases were significantly correlated with survival
Nieder C., et al., 2018, Nordland Hospital, Bodø, Norway [25]	Comparison of 2 groups of patients managed by a multidisciplinary palliative care team vs. none (36 and 65, respectively)	to analyze differences in symptom burden, baseline and outcome parameters, including completion of PRT and 30-day mortality, between PRT patients treated exclusively by regular oncology staff or a multidisciplinary palliative care team in addition (retrospective)	Failure to conclude RT was higher in the multidisciplinary palliative care team group (11 and 2%, respectively, *p* = 0.05), and also 30-day mortality was different (28 and 2%, respectively, *p* = 0.0001). Survival was not significantly different (1-year survival rates 21 and 25% respectively, *p* = 0.27)
Manfrida S., et al., 2019, Catholic University of the Sacred Heart, Rome, Italy [36]	Multidisciplinary program (ROs and anesthetists)	to provide a multidisciplinary program (ROs and anesthetists) for cancer patients with pain, to assess the IMprovement in MAnagement (IM-MA study) of this symptom (retrospective)	After 4 weeks of evaluation and interventions, inadequate pain management decreased from 27.7% to 1.5%. This data was directly correlated with age (*ρ* = 0.0297) and performance status (*ρ* = 0.0137), and inversely with RT fractionation (*ρ* = −0.0296)

*Legend*: PCC: palliative cancer care; PRT: palliative radiation therapy; ROs: radiation oncologists; RT: radiotherapy.

**Table 3:** Characteristics and main findings on studies on improved home care and outpatient status by palliative radiotherapy.

Authors, Year, Center (Ref.)	Setting	Aim (Study Design)	Main Findings
Ishii K., et al., 2021, Shizuoka cancer Center [32]	Out-patients (older than 75 years) treated with PRT due to esophageal cancer-related dysphagia	to assess the duration of survival as outpatients after RT alone (retrospective)	Median survival: 14 months. Median outpatient care: 9 months
Cellini F., et al., 2021, Catholic University of the Sacred Heart, Rome, Italy [17]	Summary of available clinical guidelines (13 papers) for PRT during COVID-19 pandemic proposed by AIRO members	to propose a clinical care model (NORMALITY) collecting available clinical guidelines for PRT during COVID-19 pandemic (systematic review)	2 levels of telemedicine-based evaluations (triage and remote visits with possibility of images sharing) are planned to decide patient’s indication to PRT

*Legend*: PRT: palliative radiation therapy; AIRO: Associazione Italiana di Radioterapia e Oncologia Clinica.

**Table 4:** Characteristics and main findings on studies about education and training of experts in supportive therapies.

Authors/Year/Ref.	Setting	Aim (Study Design)	Main Findings
Martin et al., 2020, all US Accreditation Council on Graduate Medical Education-accredited hospice and palliative medicine fellowship programs [10]	US Accreditation Council for Graduate Medical Education accredited hospice and palliative medicine fellowship programs (19-item anonymous questionnaire)	to evaluate the need of education on PRT among hospice and palliative medicine fellows (Cross-sectional survey)	51% did not receive any PRT education, 35% received only 1 or 2 h of PRT education, and only 14% received more than 2 h of PRT education with a rotation in RT during fellowship. 95% of participants agreed with the requirement of training in RT during hospice and palliative medicine fellowship; 96% felt that RT should be taught as a fundamental care in PCC; only 25% assessed their knowledge of RT principles as sufficient
Martin and Jones 2020 [11]	US Accreditation Council for Graduate Medical Education-accredited hospice and palliative medicine fellowship programs	to assess the need of education on PRT among hospice and palliative medicine program directors (Cross-sectional survey)	81 out of 120 program directors completed the survey (68% response rate). All stated the importance of a PRT curriculum in hospice and palliative medicine fellowship programs. However, PRT curriculum is absent in 30% of their programs, and only 14% have more than two hours of PRT education
Martin et al., 2019, University of California, San Diego [9]	5 hospice and palliative medicine fellows of a single institution participated in this course	to assess the impact of a PRT curriculum for hospice and palliative medicine fellows. The training course on PRT included 3 lessons of 1 h each and a visit to a RT service (Feasibility study)	100% response rate; before the course, all participants stated that knowledge on PRT was fundamental, but that they were not so confident. After the course, the mean score of the objective knowledge judgment was 86%. They were also surveyed after 3 months, reporting 80% as the mean score of the objective knowledge assessment. Also their confidence on PRT increased significantly

*Legend*: PCC: palliative cancer care; PRT: palliative radiotherapy; ROs: radiation oncologists; RT: radiotherapy; US: United States.

**Table 5:** Characteristics and main findings on studies about education and training of radiation oncologists.

Authors/Year/Ref.	Setting	Aim (Study Design)	Main Findings
Krishnan et al., 2017, all US RT residents [14]	US RT residents	to assess training needs and experience on PCC of US RT residents to guide future palliative oncology educational interventions (Electronic survey)	404/433 (93%) of US RT residents answered the survey; 79% of residents stated their education as “not/minimally/somewhat” adequate across all domains. Most (96%) reported PCC as an important competency within RO and 81% expressed a wish to more PCC training
Wei et al., 2017, all US RO residency programs [15]	Directors of RT residency programs in US	to evaluate palliative supportive care and PRT training curricula in RT residency programs in US (Electronic survey)	57/87 (63%) answered the survey. Palliative supportive care (93%) and PRT (99%) were considered as important competencies for RT residents and fellows; RT programs dedicate one or few more hours of didactics on management of (i) pain (67%), (ii) neuropathic pain (65%), (iii) nausea and vomiting (63%)
Wei et al., 2017, all ASTRO US practicing members [16]	4093/649 (16%) of all ASTRO US practicing members	to self-assess palliative supportive care skills and access to palliative supportive care training (Electronic survey)	4093/649 (16%) answered to the survey. 91% of ROs defined palliative supportive care as a fundamental competency. Symptoms like pain and gastrointestinal symptoms were reported as manageable, whereas anorexia, anxiety, and depression were reported as difficult to control; 42% of ROs do not receive any further palliative supportive care education beyond their residency training
Lo Presti et al., 2020, NA [12]	Literature review (19 paper extracted about ROs role, perceptions and education in PCC)	to report available literature data on role, perception and training needs in PCC of both ROs and trainees (Systematic review)	100% of all papers reported as fundamental the role of PCC in RT and: (i) need of education in PCC for ROs (89.4%); (ii) need of PCC in resident programs (68.4%); (iii) importance of trained ROs in PCC (63.1%); (iv) perception of inadequate training in PCC (52.6%); (v) need of skills in communication (63.1%) and pain management (47.3%); (vi) lack of research and PCC topics in RT meetings (36.8%); (vii) lack of guidelines on PCC approaches (21%)

*Legend*: PCC: palliative cancer care; PRT: palliative radiotherapy; ROs: radiation oncologists; RT: radiotherapy; US: United States; ASTRO American Society for Radiation Oncology; RO: radiation oncology.

## Supplementary Materials

The following supporting information can be downloaded at: https://www.mdpi.com/article/10.3390/curroncol29100627/s1, Table S1: narrative review checklist.
