# Supplementary material for: Improving the Integration between Palliative Radiotherapy and Supportive Care: A Narrative Review
Source: Curr Oncol. 2022 Oct 19;29(10):7932–42. doi: 10.3390/curroncol29100627 (PMC9601168; doi:10.3390/curroncol29100627)
Supplement: Supplementary file 1 [file curroncol-29-00627-s001.zip › curroncol-1928244-supplementary.pdf]

Current Oncology

Supplementals

Improving the Integration between Palliative Radiotherapy and Supportive Care: A Narrative Review

Erica Scirocco [1,2,*], Francesco Cellini [3,4], Costanza Maria Donati [1,2], Jenny Capuccini [5], Romina Rossi [6], Milly Buwenge [2], Luigi Montanari [5], Marco Maltoni [2,6,7] and Alessio Giuseppe Morganti [1,2]

Table S1. narrative review checklist.

Section/topic	#	Checklist item	Reported on page or line #
		**TITLE**	
Title	1	Identify the report as a Narrative Review	1
		**ABSTRACT**	
Unstructured summary	2	Provide an unstructured summary including, as applicable: background. Objective, brief summary of narrative review and implications for future research, and clinical practice or policy development.	1
		**INTRODUCTION**	
Rationale/background	3	Describe the rationale for the review in the context of what is already known	2
Objectives	4	Specify the key question(s) for the review topic	2
		**METHODS**	
Research selection	5	Specify the process for identifying the literature search (e.g., years considered, language, publication status, study design, and databases of coverage)	2
		**RESULTS/SUMMARY**	
Narrative	6	Discuss: 1) research reviewed including fundamental or key findings, 2) limitations and/or quality of research reviewed, and 3) need for future research.	2-10
Summary	7	Provide and overall interpretation of the narrative review in the context of clinical practice for health professionals, policy development and implementation, or future research.	10
